# Intermittent immunoadsorption in critically ill patients with neuroimmunological disorders: a retrospective study

**DOI:** 10.3389/fneur.2025.1666042

**Published:** 2025-11-27

**Authors:** Qian Wu, Lin Zhu, Shujuan Dai, Jing Xu, Deshan Ge

**Affiliations:** 1 Department of Neurology, First Affiliated Hospital, Kunming Medical University, Kunming, Yunnan, China; 2 Department of Rehabilitation, The Third People’s Hospital of Yunnan Province, Kunming, Yunnan, China

**Keywords:** immunoadsorption, neurocritical care, autoimmune neurological disorders, therapeutic apheresis, intensive care unit

## Abstract

**Objectives:**

This study aimed to evaluate the efficacy and safety of intermittent immunoadsorption (IA) in critically ill patients with refractory autoimmune neurological disorders.

**Methods:**

We retrospectively reviewed 13 patients admitted to the neurocritical care unit with severe autoimmune encephalitis, Guillain–Barré syndrome, neuromyelitis optica spectrum disorders, or chronic inflammatory demyelinating polyneuropathy, all of whom had failed first-line immunotherapy (intravenous methylprednisolone and/or intravenous immunoglobulin). IA was administered intermittently, with schedules individualized based on clinical status.

**Results:**

The modified Rankin Scale (mRS) improved significantly following IA (*p* = 0.02), while the Acute Physiology and Chronic Health Evaluation II scores (APACHE II) remained stable (*p* = 0.95). Serum IgG levels declined by a median of 55.6%. Pathogenic antibody negativity was achieved in 65% of plasma and 38% of cerebrospinal fluid samples. Although 92% experienced treatment interruptions (e.g., infection and hypotension), IA was generally well tolerated and not permanently discontinued.

**Discussion:**

This study supports the feasibility and clinical utility of intermittent IA in critically ill patients with treatment-refractory neuroimmunological disorders. Despite frequent complications, flexible scheduling allowed continued therapy with sustained benefit. These findings highlight a potentially adaptable treatment strategy in a population often excluded from therapeutic interventions and suggest that IA warrants further study in neurocritical care settings.

## Introduction

Immunoadsorption (IA) is a therapeutic apheresis technique designed to remove pathogenic antibodies from circulation with high specificity (1). It has proven efficacy in immune-mediated neurological diseases such as autoimmune encephalitis (AE), myasthenia gravis, neuromyelitis optica spectrum disorders (NMOSDs), and Guillain–Barré syndrome (GBS) (1). In less critically ill patients, IA is generally well tolerated and associated with favorable clinical outcomes (2). However, its use in critically ill patients remains underreported, limited by concerns about safety, immunosuppression, and procedural risk. This retrospective study evaluates the feasibility, tolerability, and clinical outcomes of intermittent IA in critically ill patients with refractory immune-mediated neurological diseases, aiming to address this therapeutic gap.

## Methods

### Design

This retrospective observational study included 13 critically ill patients admitted to the neurocritical care unit (NCU) between January 2021 and July 2024 who received intermittent IA for refractory autoimmune neurological disease. Inclusion criteria included: (1) confirmed diagnosis, (2) failure of first-line treatment (intravenous methylprednisolone (IVMP) and/or intravenous immunoglobulin (IVIG)), and (3) clinical deterioration requiring intensive care (e.g., respiratory failure and severe motor deficits). Patients with mild disease (Acute Physiology and Chronic Health Evaluation II (APACHE II) < 5 or Modified Rankin Scale (mRS) < 2) were excluded. Both baseline scores were assessed upon admission to the NCU. All eligible patients during the study period were included consecutively to reduce selection bias. Standardized measures (mRS, APACHE II) and certified laboratory tests were used to minimize observer and information bias. No sample size calculation was performed due to the retrospective nature. IA was performed using a protein A adsorber (KONPIA®; Guangzhou Koncen Bioscience Co., Ltd., Guangzhou, China) and a hemoperfusion device (DTB-100A, Chongqing Duotai Medical Device Co., Ltd., Chongqing, China). Sessions were adapted based on patient tolerance, infection status, and immune function. The standard operating procedure for IA used at our center is provided as Supplementary materials.

### Measurements

Demographic and clinical data were derived from the review of medical records (Table 1), including neurological and disease severity assessments, as well as IA-related parameters (Table 2). General outcomes included the mRS scale and APACHE II score at admission and discharge, respectively. The mRS is a clinically validated functional assessment scale (0–6), where 0 = no symptoms, 1–2 = mild disability, 3–4 = moderate to severe disability, 5 = severe disability requiring constant care, and 6 = death. A change of ±1 point was considered deterioration/improvement (3). Disease severity was assessed using the APACHE II score (4), which ranges from 0 to 71, with higher scores indicating greater physiological derangement and increased mortality risk. IA treatment outcomes included serum IgG levels pre- and post-each IA session and pathogenic antibody alterations in plasma and cerebrospinal fluid (CSF), classified as negative conversion (undetectable after treatment), reduction (decrease in antibody titer but still detectable), and no change (persistent antibody detection).

**Table 1:** Individual patient characteristics before and after IA treatment.

#	Diag	Clinical presentation	Previous immunotherapy	IA ses	Antibody					mRS		APACHE		NCU Days	MV Days	Adverse Events
					Type	Plasma		CSF		Adm	∆	Adm	∆			
						Adm	Dis	Adm	Dis							
1	AE	Syncope, behavioral disorder, seizures	IVIG, IVMP	4	NMDAR	1:10	−	1:10	−	3	1	9	0	35	0	*Hypotension*; chills
2	AE	Agitation, apnea	IVIG, IVMP	7	NMDAR	1:10	−	1:10	1:10	5	1	6	−3	59	58	*Hypotension*
3	AE	Headache, behavioral disorder	IVIG, IVMP	3	NMDAR	1:320	1:10	1:32	1:32	4	1	9	−5	21	0	*Hypotension*
4	AE	Impaired consciousness, seizures	IVIG, IVMP	6	mGLU5	1: 32	1: 1	1: 32	1: 10	5	0	25	4	79	79	Sepsis; *leukemoid reaction; hypotension*
5	AE	Seizures	IVMP	3	anti-GM4	+	−	−	−	2	1	9	−5	16	0	*Hypotension*
					CASPR2	1:32	−	−	−							
6	GBS	Diplopia, dyspnea	IVIG, IVMP	10	MAG	1:10	−	−	−	5	0	7	−2	90	84	*Hypotension*
7	GBS	Limb weakness	IVIG, IVMP	10	GM1	+	−	−	−	5	0	12	5	65	62	Catheter related infection; *abdominal pain*
					anti-GM4	+	+	−	−							
					GD1b	+	−	−	−							
8	GBS	Limb weakness, dyspnea	IVIG	7	GM1	+	−	−	−	5	3	15	10	16	10	Rash on the chest
					GQ1a	+	−	−	−							
9	GBS	Behavioral disorder	IVIG, IVMP	2	GQ1b	+	+	+	−	3	2	12	7	24	15	OB (+), Hb 57 g/L; pelvic hematoma
10	CIPD	SOB, limb weakness	IVIG	5	NF186	+	−	−	−	4	2	11	2	41	30	/
11	NMOSDs	Numbness, limb weakness	IVIG, IVMP	10	AQP4	1:10	1: 10	1:3.2	1: 1	4	0	10	0	23	0	*Headache*
12	NMOSDs	Numbness, limb weakness	IVIG, IVMP	10	AQP4	1: 1000	1: 10	1: 1000	1: 320	4	0	5	−4	70	65	Thymectomy; Sepsis; *hypotension*
13	NMOSDs	Vomiting, numbness, limb weakness, dyspnea	IVIG	9	AQP4	1:10	−	1:10	−	5	0	14	−6	61	61	Cardiac arrest; *hypotension*

“Diag”, diagnosis; “IA Ses”, immunoadsorption sessions; “Adm”, admission, “Dis”, discharge; “∆”, change from admission to discharge; “MV”, mechanical ventilation. Immunotherapy abbreviations: IVIG, intravenous immunoglobulin; IVMP, intravenous methylprednisolone Disease abbreviations: AE, autoimmune encephalitis; GBS, Guillain–Barré syndrome; CIDP, chronic inflammatory demyelinating polyradiculoneuropathy; NMOSDs, neuromyelitis optica spectrum disorders; Medical and antibodies’ abbreviations: SOB, shortness of breath; CSF, cerebral spinal fluid; NCU, neurocritical unit; NMDAR, N-methyl-D-aspartate receptors; mGLU5, metabotropic glutamate receptor 5; anti-GM4, antiganglioside; CASPR2, contactin-associated protein-like 2; MAG, myelin-associated glycoprotein; GM1/GM4, GD1b, and GQ1a/GQ1b are gangliosides; NF186, neurofascin-186; AQP4, aquaporin-4; “–” indicates a negative antibody result. Adverse events shown in italics represent transient events that occurred repeatedly during IA procedures.

**Table 2:** Individual IA treatment characteristics.

No.	Diagnosis	Parameters	IA sessions									
			1	2	3	4	5	6	7	8	9	10
1	AE	Cycles	6	6	6	6	/	/	/	/	/	/
		Total circulation (ml)	3,600	3,600	3,600	3,600	/	/	/	/	/	/
		ISIs (days)	-	1	0	2	/	/	/	/	/	/
		Pre-IgG (g/L)	5.69	8.11	5.43	2.73	/	/	/	/	/	/
		Post-IgG (g/L)	1.01	1.25	0.72	0.4	/	/	/	/	/	/
2	AE	Cycles	8	8	5	5	5	5	6	/	/	/
		Total circulation (ml)	4,800	4,800	3,000	3,000	3,000	3,000	3,600	/	/	/
		ISIs (days)	-	0	25	1	3	1	1	/	/	/
		Pre-IgG (g/L)	17.1	7.43	8.78	7.8	14.5	17.4	14.9	/	/	/
		Post-IgG (g/L)	7.33	2.14	7.11	5.93	9.64	10.6	9.44	/	/	/
3	AE	Cycles	8	8	8	/	/	/	/	/	/	/
		Total circulation (ml)	4,800	4,800	4,800	/	/	/	/	/	/	/
		ISIs (days)	0	0	0	/	/	/	/	/	/	/
		Pre-IgG (g/L)	4.6	3.33	3.9	/	/	/	/	/	/	/
		Post-IgG (g/L)	1.65	1.14	1.29	/	/	/	/	/	/	/
4	AE	Cycles	10	10	10	8	8	8	/	/	/	/
		Total circulation (ml)	6,000	6,000	6,000	4,800	4,800	4,800	/	/	/	/
		ISIs (days)	-	0	12	0	2	1	/	/	/	/
		Pre-IgG (g/L)	6.06	4.16	11.6	7.33	6.39	8.07	/	/	/	/
		Post-IgG (g/L)	1.19	1.37	5.84	2.82	6.52	7.23	/	/	/	/
5	AE	Cycles	8	8	8	/	/	/	/	/	/	/
		Total circulation (ml)	4,800	4,800	4,800	/	/	/	/	/	/	/
		ISIs (days)	-	0	0	/	/	/	/	/	/	/
		Pre-IgG (g/L)	7.37	DNT	3.23	/	/	/	/	/	/	/
		Post-IgG (g/L)	3.56	DNT	1.01	/	/	/	/	/	/	/
6	GBS	Cycles	10	8	8	8	8	8	10	10	10	8
		Total circulation (ml)	6,000	4,800	4,800	4,800	4,800	4,800	6,000	6,000	6,000	4,800
		ISIs (days)	0	4	1	1	3	0	9	1	2	1
		Pre-IgG (g/L)	10	7.46	5.11	7.66	DNT	6.06	11.5	8.58	7.73	9.78
		Post-IgG (g/L)	2.24	1.82	4.14	DNT	1.95	1.75	5.7	4.86	4.31	6.64
7	GBS	Cycles	8	8	8	8	8	8	8	8	8	8
		Total circulation (ml)	4,800	4,800	4,800	4,800	4,800	4,800	4,800	4,800	4,800	4,800
		ISIs (days)	-	0	0	2	1	1	2	1	1	1
		Pre-IgG (g/L)	16.8	11.2	6.48	6.76	4.32	2.49	3.23	3.37	3.23	3.04
		Post-IgG (g/L)	10.3	DNT	6.82	3.12	1.89	1.28	1.52	1.29	1.22	1.18
8	GBS	Cycles	8	8	8	8	5	8	8	/	/	/
		Total circulation (ml)	4,800	4,800	4,800	4,800	3,000	4,800	4,800	/	/	/
		ISIs (days)	-	0	0	2	1	2	0	/	/	/
		Pre-IgG (g/L)	17.3	8.09	10.04	5.87	7.43	DNT	8.21	/	/	/
		Post-IgG (g/L)	8.09	DNT	4.22	1.79	3.16	DNT	3.91	/	/	/
9	GBS	Cycles	8	8	/	/	/	/	/	/	/	/
		Total circulation (ml)	4,800	4,800	/	/	/	/	/	/	/	/
		ISIs (days)	-	0	/	/	/	/	/	/	/	/
		Pre-IgG (g/L)	17.2	6.01	/	/	/	/	/	/	/	/
		Post-IgG (g/L)	6.01	0.95	/	/	/	/	/	/	/	/
10	CIPD	Cycles	N/A	10	10	10	10	/	/	/	/	/
		Total circulation (ml)	N/A	6,000	6,000	6,000	6,000	/	/	/	/	/
		ISIs (days)	N/A	0	30	0	0	/	/	/	/	/
		Pre-IgG (g/L)	25.3	10.07	16.9	14.6	17.3	/	/	/	/	/
		Post-IgG (g/L)	18	6.98	DNT	DNT	DNT	/	/	/	/	/
11	NMOSDs	Cycles	8	8	8	8	8	8	8	8	8	8
		Total circulation (ml)	4,800	4,800	4,800	4,800	4,800	4,800	4,800	4,800	4,800	4,800
		ISIs (days)	-	0	1	1	1	4	2	0	2	0
		Pre-IgG (g/L)	16.7	17.8	10.6	7.8	10.4	14.8	12.5	6.82	13.9	11.1
		Post-IgG (g/L)	7.84	8.53	DNT	2.24	3.9	8.41	6.28	5.53	6.29	6.24
12	NMOSDs	Cycles	8	8	8	4	8	4	8	10	8	10
		Total circulation (ml)	4,800	4,800	4,800	2,400	4,800	2,400	4,800	6,000	4,800	6,000
		ISIs (days)	-	0	0	0	2	0	3	3	2	3
		Pre-IgG (g/L)	29.2	14.3	6.83	3.46	8.56	3.68	11.5	9.51	6.27	8.47
		Post-IgG (g/L)	13.6	5.99	1.99	1.72	1.9	1.82	4.25	1.94	1.59	2.61
13	NMOSDs	Cycles	5	5	5	5	5	5	5	5	5	/
		Total circulation (ml)	3,000	3,000	3,000	3,000	3,000	3,000	3,000	3,000	N/A	/
		ISIs (days)	-	0	2	2	2	1	1	11	1	/
		Pre-IgG (g/L)	20.5	17.9	11.9	17.1	13.7	9.84	16.1	12.5	14.1	/
		Post-IgG (g/L)	12.1	13.6	8.22	DNT	8.28	5.52	8.45	9.35	8.08	/

“N/A” = not applicable; “DNT” = did not test; pre/post-IgG = immunoglobulin G levels before and after each IA session; ISIs = inter-session intervals (days between each IA session); each IA session consisted of multiple cycles (the number of cycles and total circulation volume varied based on individual patient tolerance); disease abbreviations are defined in the footnote of Table 1.

### Statistical analysis

Data were analyzed using GraphPad Prism version 10.0 or later. Variables were presented as median and interquartile range (IQR). Changes in mRS and APACHE II were analyzed using the Wilcoxon test; changes in IgG were analyzed using multiple Wilcoxon tests. A *p*-value of < 0.05 was considered statistically significant.

## Results

**Figure 1:**
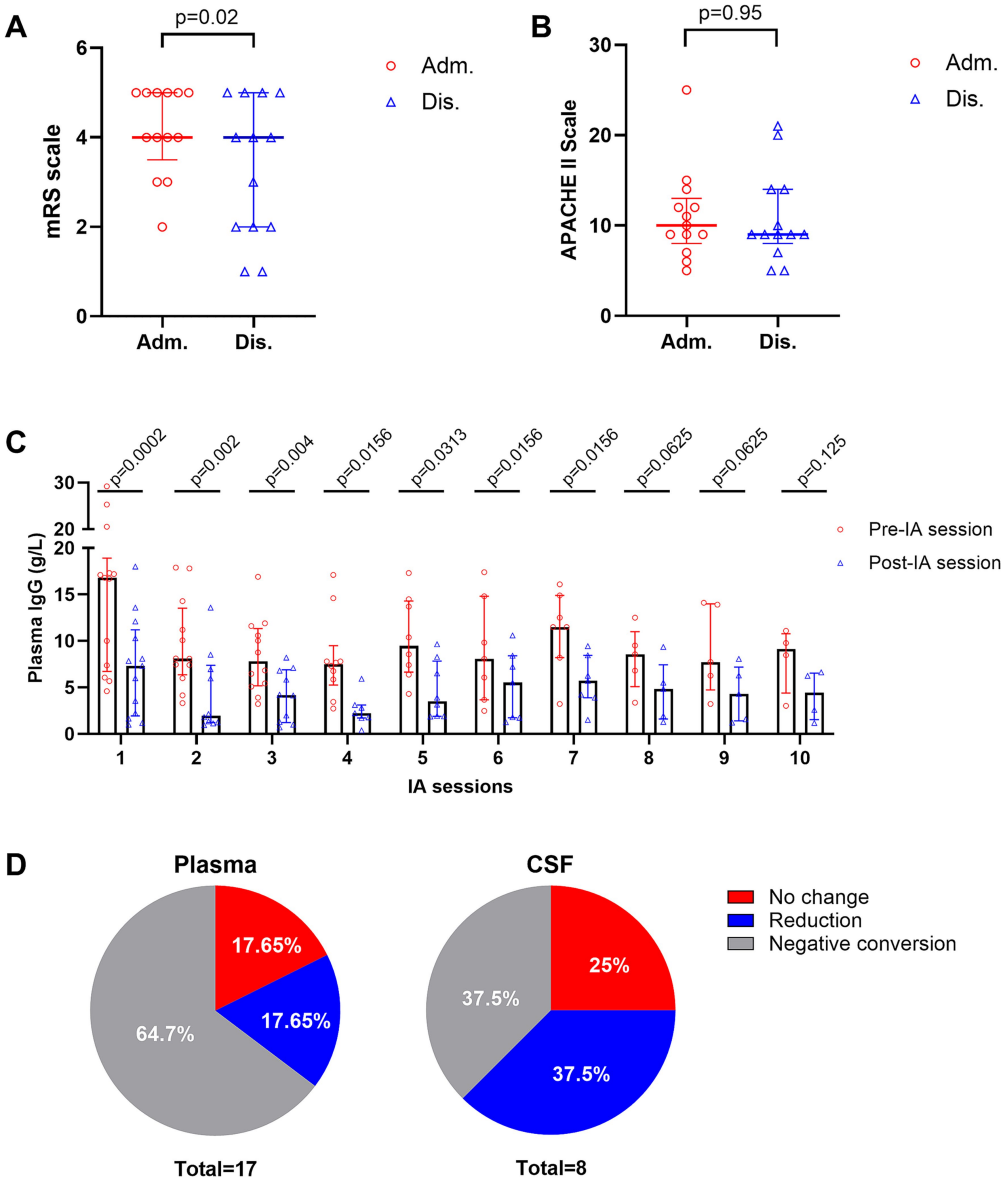
Clinical and laboratory outcomes of intermittent immunoadsorption (IA). **(A)** Individual patients’ mRS scores at admission and discharge. **(B)** APACHE II scores admission and discharge. **(C)** Changes in serum IgG levels before and after each IA session. **(D)** Pathogenic antibody responses in plasma (left) and CSF (right). Data are presented as medians with interquartile ranges.

Thirteen patients (9 women, 4 men, median age 46 years, IQR: 14–58, median body weight 70 kg, IQR: 40–80) met the inclusion criteria. Diagnoses include AE (n = 5), GBS (n = 4), neuromyelitis optica spectrum disorders (NMOSDs; n = 3), and chronic inflammatory demyelinating polyneuropathy (CIDP; n = 1). Although the median mRS score at both admission and discharge was 4 (IQR: 2–5 at admission and 1–5 at discharge), the distribution of individual changes showed a statistically significant improvement (*p* = 0.02) (Figure 1A). Specifically, 7 out of 13 patients demonstrated a ≥ 1 point reduction in mRS by discharge (Table 1), indicating meaningful functional recovery. The median APACHE II score was 10 (IQR: 5–25) on admission and 9 (IQR: 5–21) on discharge (*p* = 0.95), indicating a stable systemic condition (Figure 1B). The median duration of mechanical ventilation was 30 days (IQR: 0–62), and the median NCU stay was 41 (IQR: 23–65) days.

The median number of IA sessions per treatment course was 7 (IQR: 4–10), with inter-session intervals (ISIs) ranging from 1 to 30 days. Interruptions occurred in 92% of patients due to infection, hypotension, leukemoid reaction, or procedural complications (e.g., cardiac arrest and pelvic hematoma). Median IgG level decreased from 8.56 g/L (IQR: 6.17–14) to 4.18 g/L (IQR: 1.78–7.14), representing a median 55.6% reduction (Figure 1C, Table 2). In some cases, at our center, IVIG was administered during ISIs when serum IgG levels fell below 4 g/L, with or without concomitantly low IgA levels (~0.8 g/L), to support immunity (5). Pathogenic antibody analysis revealed negative conversion in 64.7% (plasma) and 37.5% (CSF), partial reduction in 17.65% (plasma) and 37.5% (CSF), and no change in 17.65% (plasma) and 25% (CSF) (Figure 1D).

## Discussion

Our findings suggest that intermittent IA treatment is both feasible and effective in critically ill patients with immune-mediated neurological disorders who fail standard first-line therapies. The flexible schedule allows clinicians to pause IA during critical complications and restart it when stable. In our center, decisions to suspend or prolong ISIs were based on multiple factors, including overall patient tolerance and the occurrence of serious adverse events (e.g., hypotension requiring vasopressor support, sepsis, or catheter-related infection). Among these, infectious complications typically result in substantially longer ISIs. This adaptive model may improve safety without compromising efficacy.

Notably, neurological function, as assessed by mRS, improved in most patients, whereas systemic disease severity, as measured by the APACHE II score, remained stable despite clinical complications. Given that the APACHE II score incorporates parameters from multiple organ systems, it provides an objective measure of overall physiological derangement (4). These findings suggest that intermittent IA treatment is a safe treatment option for critically ill patients with autoimmune neurological disorders.

Minor complications commonly associated with apheresis and vascular access (e.g., transient hypotension and hematoma) were observed in our cohort, although absent in some prior reports (6), and were clinically manageable. Leukemoid reactions were transient and self-limiting, while catheter-related infections and sepsis were successfully treated and did not necessitate permanent discontinuation of IA therapy. One patient experienced cardiac arrest during IA but was successfully resuscitated with cardiopulmonary resuscitation and completed the planned treatment course. In contrast, the reported adverse events in the intensive care unit during plasma exchange included anaphylactoid reactions, severe hypotension, catheter-related infections, pneumothorax, local bleeding, hypocalcemia, and paresthesia (7). These complications, largely related to plasma substitution and citrate anticoagulation, were uncommon in our IA cohort (Table 1), suggesting that IA may offer a favorable safety profile in critically ill neurologic patients, although confirmation in larger prospective studies is warranted.

While our observed IgG reduction was slightly lower than previously reported in general populations (55.6% vs. 62–93%) (1), this may reflect lower starting IgG levels or treatment interruptions. Despite concerns about IA-related immunosuppression, no patient in our cohort experienced fatal infection or irreversible deterioration. This supports the safe use of IA in selected patients with marginal IgG values, provided monitoring and IVIG support are available.

IA remains underutilized, partly due to high costs and limited guidelines for ICU settings. In Germany, a session costs ~€ 1,200 (8); in the U. S., five sessions may cost ~$ 58,952 (9). At our center, a typical course costs ~30,000 RMB (~3,000 per session). Costs vary widely based on location, condition, and institutional procurement, but early use in high-risk patients may reduce long-term ICU burden.

Our study has several limitations. First, its retrospective design introduces inherent biases, including a small sample size and the absence of a control group. Additionally, the study was not powered to detect differences across subgroups of immune-mediated neurological diseases. The absence of certain severe conditions, such as myasthenic crisis, further limits the generalizability of our findings. Future research should focus on prospective, controlled studies with multiple centers and large cohorts to validate our observations and explore the optimal IA treatment protocols for diverse neuroimmunological conditions.

## Data Availability

The raw data supporting the conclusions of this article will be made available by the authors, without undue reservation.
