# Supplementary material for: Intermittent immunoadsorption in critically ill patients with neuroimmunological disorders: a retrospective study
Source: Front Neurol. 2025 Nov 27;16:1666042. doi: 10.3389/fneur.2025.1666042 (PMC12695613; doi:10.3389/fneur.2025.1666042)
Supplement: Supplementary file 1 [file Data_Sheet_1.pdf]

Supplementary material

Standard Operating Procedure for Protein A Immunoadsorption (IA) in the Neurocritical Care Unit

1.Purpose

To outline the standardized process for performing protein A–based immunoadsorption (IA) in critically ill patients with autoimmune neurological disorders at the First Affiliated Hospital of Kunming Medical University, ensuring safety, reproducibility, and optimal antibody removal.

2. Patient Preparation

- Pre-treatment laboratory tests: Complete blood count, electrolytes, coagulation profile, immunoglobulin quantification, and disease-related antibodies.
- Consent: Obtain informed consent for IA therapy.
- Vascular access: Insert a temporary ≥12F double-lumen central venous catheter (internal jugular or femoral vein) 24 hours before the first IA session to allow hemostasis and ensure patency.
- Baseline assessment: Record body weight, vital signs, and antibody titers.

3. Contraindications

- Pregnancy.
- Inability to cooperate.
- Unconsciousness without airway protection.
- Relative contraindications to extracorporeal circulation: shock, hypercoagulable state, severe bleeding tendency, uncontrolled infection, or cardiorespiratory failure.
- Known intolerance or allergy to plasma exchange or extracorporeal therapy.
- Uncontrolled hypertension.
- Multi-organ failure.
- Severe anxiety or agitation.

4.Procedure Workflow

a. Verify patient identity, consent, and laboratory results.
Commercially available protein A adsorption columns and standard extracorporeal apheresis equipment were used according to the manufacturer's instructions.

b. Procedure sequence:
Cycle: Priming → Adsorption → Plasma return → Elution → Equilibration → Re-priming → Repeat adsorption. *Cycles were repeated until the target plasma volume (approximately 3–6 L or 8-10 cycles, corresponding to about 1–1.5 times the patient's estimated plasma volume) was achieved*.

*Plasma volume (mL) can be estimated using the formula:

$$\text{Plasma volume (mL)} = 65 \times \text{body weight (kg)} \times (1 - \text{hematocrit})$$

Detailed steps:

1) Column priming:
   - Flush the protein A adsorption column sequentially with 0.9% normal saline (2,500 mL), heparinized saline (15 mg heparin in 500 mL), and 0.9% normal saline (500 mL).
   - Flow rate: 60 mL/min.
   - Prime the plasma separator in parallel.

2) Adsorption:
   - Blood-pump rate: 125 mL/min.
   - Plasma-pump rate: 35 mL/min (not > 30% of blood-flow rate).
   - Duration: 15 min.

3) Plasma return:
   - Plasma-pump rate: 60 mL/min; duration: 3 min.

4) Elution:
   - Plasma-pump rate: 60 mL/min; duration: 7 min.
   - Target pH 2.2–2.8 to complete elution.

5) Equilibration:
   - Plasma-pump rate: 60 mL/min; duration: 7 min.
   - Target pH ≈ 7.0 to complete equilibration.

6) Re-priming:
   - Flush with saline at 60 mL/min for 3 min before resuming adsorption.

c. Final termination criteria:

IA treatment course was concluded when (1) clinical improvement was achieved, (2) disease-associated antibodies converted to negative, or (3) the planned 10 sessions had been completed—whichever occurred first.

5. Safety and Troubleshooting

All IA sessions were conducted under continuous hemodynamic and oxygen-saturation monitoring. Common procedure-related adverse effects included transient hypotension, mild allergic reactions, and catheter-related complications, all of which were self-limited or managed with supportive care.

Treatment was paused or the inter-session interval (ISI) extended in cases of serious events such as hypotension requiring vasopressor support, sepsis, catheter-related infection, or cardiac arrest. The procedure was resumed once the patient's condition stabilized.

6. Documentation

- Record session number, total circulation volume, ISI, pre-/post-IgG levels, antibody titers (if possible), and complications in the IA log sheet.
- Report any severe or life-threatening events to the attending physician immediately.

** This SOP represents the standard intermittent IA protocol applied at our center (Neurocritical Care Unit in First Affiliated Hospital, Kunming Medical University). Individual modifications (e.g., inter-session interval extension) were made according to patient tolerance and clinical condition.*
